# Cytoplasmic anion/cation imbalances applied across the membrane capacitance may form a significant component of the resting membrane potential of red blood cells

**DOI:** 10.1038/s41598-022-19316-z

**Published:** 2022-09-02

**Authors:** Michael Pycraft Hughes, Christopher H. Fry, Fatima H. Labeed

**Affiliations:** 1 Department of Biomedical Engineering, Khalifa University, Abu Dhabi, United Arab Emirates; 2 Centre for Biomedical Engineering, University of Surrey, Guildford, GU2 7XH Surrey UK; 3 School of Physiology, Pharmacology and Neuroscience, University of Bristol, Bristol, BS8 1TD UK

**Keywords:** Biophysics, Membrane biophysics

## Abstract

Electrical aspects of cell function manifest in many ways. The most widely studied is the cell membrane potential, *V*_*m*_, but others include the conductance and capacitance of the membrane, the conductance of the enclosed cytoplasm, as well as the charge at the cell surface (an electrical double layer) producing an extracellular electrical potential, the *ζ*-potential. Empirical relationships have been identified between many of these, but not the mechanisms that link them all. Here we examine relationships between *V*_*m*_ and the electrical conductivities of both the cytoplasm and extracellular media, using data from a suspensions of red blood cells. We have identified linear relationships between extracellular medium conductivity, cytoplasm conductivity and *V*_*m*_. This is in contrast to the standard model of a resting membrane potential which describes a logarithmic relationship between *V*_*m*_ and the concentration of permeable ions in the extracellular medium. The model here suggests that *V*_*m*_ is partially electrostatic in origin, arising from a charge imbalance at an inner electrical double-layer, acting across the membrane and double-layer capacitances to produce a voltage. This model describes an origin for coupling between *V*_*m*_ and ζ, by which cells can alter their electrostatic relationship with their environment, with implications for modulation of membrane ion transport, adhesion of proteins such as antibodies and wider cell–cell interactions.

## Introduction

Every living cell functions by partitioning ions across its cell membrane, producing for example differential quantities of K^+^, Cl^−^ and Na^+^ in its inner space (cytoplasm) compared to its surrounding extracellular medium - a so-called Donnan effect, sustained by active transport of ions. The different concentrations of each ion on either side of the membrane produces a voltage—the membrane potential, *V*_*m*_—which plays a crucial role in cell function. For example, in the case in nerve and muscle cells depolarisation of *V*_*m*_ is used as a means of inter-communication and contractile generation and the study of these cells gave rise to the present model describing the origin of *V*_*m*_^1–3^.

The search for the basis of electrical phenomena in physiology was investigated through the 19th and early twentieth centuries by such luminaries as Carlo Matteucci, Emil du Bios Raymond and Julius Bernstein^4^. Several models were developed to describe analytically the relationship between transmembrane potential differences and extracellular and intracellular ion concentrations, including the “constant field model”^5^, the “fixed surface charge” model^6^ and the semiconductor-based “junction model”^7^. All were approximations to different degrees and approached the issue in various ways. Ultimately, the adopted paradigm was a “constant field model”^8^ that described a potential difference due to a non-equilibrium steady-state between electrical and concentration gradients—with the contribution from different ions dependent on their individual permeabilities. This approach suggests that the membrane potential, *V*_m_, arises from a diffusion potential across the membrane, and represents the voltage that must be applied to the membrane in order to reduce transmembrane current to zero. The Goldman-Hodgkin-Katz (GHK) equation remains the cornerstone of cell electrophysiology. It is a remarkably useful tool to interpret non-equilibrium behaviour in membrane transport^9^ and is accepted as the de facto descriptor of *V*_m_ to interpret voltage- and current-clamp data and determine the ionic basis of electrophysiological phenomena. However, the description provided by the GHK equation is incomplete. By definition, a potential that arises purely from diffusion across a membrane has no value beyond that membrane, so that the electric field beyond the membrane is zero. In general, the GHK equation is used to interpret the role and function of membrane-based ion channels, but does not include other, equilibrium mechanisms of electrical behaviour.

**Figure 1:**
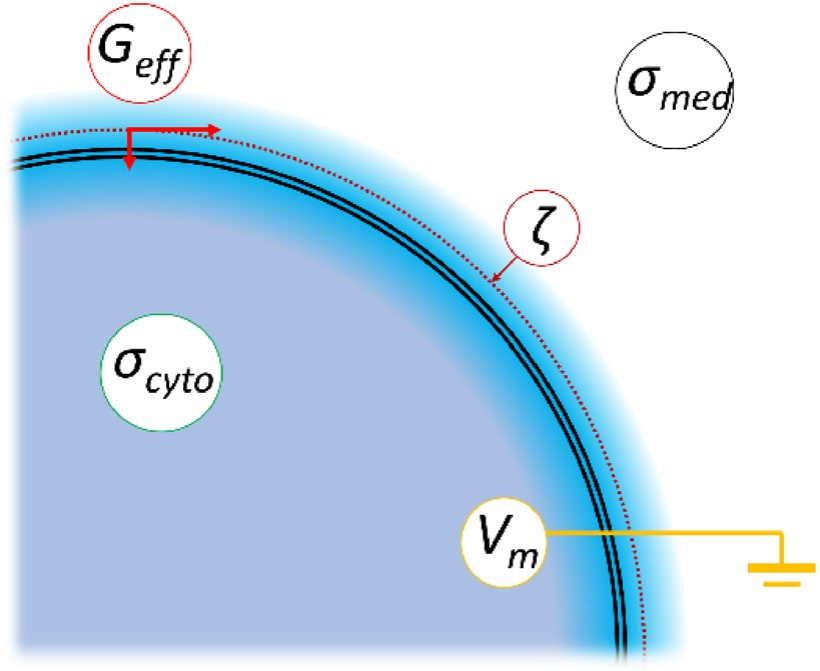
A schematic showing some of the parameters and variables used in this study. Parameters σ_med_ and σ_cyto_ represent the medium conductivity and cytoplasm conductivity respectively; membrane potential *V*_m_ represents the electrical potential inside the cell with respect to the bulk medium; *ζ* is the zeta-potential at the hydrodynamic plane of shear; and *G*_eff_ is the effective membrane conductance which comprises both transmembrane and double-layer conductances.

There is increasing evidence that *V*_*m*_ has effects beyond the membrane and into extracellular and intracellular spaces. For example, Tyner et al.^10^ used “nanosized voltmeter” nanoparticles to map the intracellular influence of *V*_m_ and observed “E fields extending out much farther (microns) into the cytosol”. Furthermore, recent observations of red blood cells (RBCs) have indicated that the extracellular *ζ*-potential (a voltage field that arises from a cell surface charge and extends several nanometres from the cell surface) is affected by *V*_*m*_, via a process related to the capacitance of the electrical double-layer^11^. Similar phenomena have also been identified in platelets^12^. Connections have also been identified between *V*_*m*_ and passive cellular electrical properties, such as the cytoplasm conductivity *σ*_*cyto*_ and the membrane conductance *G*_*eff*_^13–15^. For example, studies of electrophysiological circadian rhythms identified highly synchronised patterns of behaviour in cytoplasm conductivity, *σ*_*cyto*_, the effective membrane conductance, *G*_*eff*_, *V*_*m*_, and the *ζ-*potential^15,16^. The synchronicity of these phenomena (a combination of both in-phase and antiphase relationships) strongly suggests that these variations are different manifestations of a common underlying process, though not what such a process might be. Whilst connections appear to be generalisable across several cell types, the mechanism remains unknown. A schematic showing these different electrical properties is shown in Fig. 1.

To identify a common mechanism, we consider here the inter-dependence of *V*_*m*_, *σ*_*cyto*_, and the medium conductivity, *σ*_*med*_. Expressions are identified that relate these properties across different experimental conditions, designed to alter the medium composition or cell membrane surface charge. Key relationships are identified which suggest a strong causal relationship between many of these properties, together with additional, inferred parameters such as the outer and inner membrane surface potentials. These relationships suggest that *σ*_*cyto*_ is largely driven by diffusion and an established Donnan equilibrium, and that *V*_*m*_ is capacitively coupled across the membrane, with the ensuing induced charge potentially altering both the ζ-potential and surface membrane conductance.

## Theory

The standard model of *V*_*m*_ derives from the theory that a semi-permeable membrane gives rise to different intracellular and extracellular concentrations of permeable ions such as K^+^. Bernstein produced a diffusion-based model, built on the more generalised work of Nernst:

1 $${V}_{m}=-\frac{RT}{F}ln\left(\frac{{K}_{in}}{{K}_{out}}\right)$$

where *R*, *F* and *T* have their usual thermodynamic meanings, and the suffixes *in* and *out* refer to intracellular and extracellular K^+^ concentrations. The model was refined for generalised biological membranes and applied to excitable cell function by including the influence of Na^+^ and Cl^−^^5,8^:

2 $${V}_{m}=\frac{RT}{F}ln\left(\frac{{P}_{{Na}^{+}}{\left[{Na}^{+}\right]}_{out}+ {P}_{{K}^{+}}{\left[{K}^{+}\right]}_{out}+{P}_{{Cl}^{-}}{\left[{Cl}^{-}\right]}_{in} }{{P}_{{Na}^{+}}{\left[{Na}^{+}\right]}_{in}+ {P}_{{K}^{+}}{\left[{K}^{+}\right]}_{in}+{P}_{{Cl}^{-}}{\left[{Cl}^{-}\right]}_{out}}\right)$$

where *P*_*x*_ describes the permeability of each ion (*x*) across the membrane. This GHK Equation describes a steady-state situation for monovalent ions, although it may be adapted for multivalent ions with significant membrane permeabilities such as Ca^2+^ ^16^.

Other methods can be applied to different aspects of the electrical state of a cell. For example, dielectrophoresis (DEP), the induced motion of polarisable particles suspended in non-uniform electric fields^17^, has been used to determine the dielectric properties of cells. This is typically performed by measuring variation of the DEP force as a function of frequency and relating this to variations in the Clausius–Mossotti factor, *K*(*ω*)^18^, which yields values for membrane conductivity and capacitance, and intracellular conductivity, thus:

3 $$K(\omega )=\frac{{\varepsilon }_{cell}^{*}-{\varepsilon }_{med}^{*}}{{\varepsilon }_{cell}^{*}+2{\varepsilon }_{med}^{*}}$$

4 $${\varepsilon }_{cell}^{*}={\varepsilon }_{m}^{*}\frac{{\left(\frac{r+t}{r}\right)}^{3}+2\frac{{\varepsilon }_{c}^{*}-{\varepsilon }_{m}^{*}}{{\varepsilon }_{c}^{*}+2{\varepsilon }_{m}^{*}}}{{\left(\frac{r+t}{r}\right)}^{3}-\frac{{\varepsilon }_{c}^{*}-{\varepsilon }_{m}^{*}}{{\varepsilon }_{c}^{*}+2{\varepsilon }_{m}^{*}}}$$

where ε* is the complex permittivity,

5 $${\varepsilon }^{*}=\varepsilon -j\frac{\sigma }{\omega }$$

Here *ε* is the permittivity, *σ* the conductivity, *ω* the angular frequency of the applied field and *j* the complex operator √−1; *r* is the cell radius and *t* the membrane thickness; the subscript *cell* refers to the whole cell, *med* to the suspending medium *c* to the cytoplasm and *m* to the membrane.

However, this approach to determining cell properties is somewhat reductive; for example, the “membrane conductance” parameter is actually a composite of transmembrane conductance and surface conductance through an electrical double-layer^19–22^. Furthermore, the DEP response yields only conductivity and permittivity data, rather than directly measuring *V*_m_.

Another group of important electrical parameters are potentials arising from charges on the cell surface. These charges generate surface potentials (*ψ*) which then attract counter-charges from the surrounding ionic solution; e.g. a negatively charged surface will attract cations and repel anions. This will lead to charge imbalances in the volume immediately surrounding the surface, known as the electrical double-layer. There are several standard models describing ion concentrations outside a charged surface^23^. These can be summarised as comprising a layer immediately outside the membrane, composed of ions and dipolar solvent molecules that are electrostatically immobile at the surface (the Stern layer), surrounded by a layer of diffuse ion imbalance (the Debye layer) extending into the bulk solution, with diminishing differences in anion and cation levels at increasing distance from the surface. Precise determination of parameters in such a model is problematical; for example, it is difficult to measure the surface charge and associated potential directly, leading most researchers to instead study the potential at the hydrodynamic shear plane present within the Debye layer^23^, known as the *ζ-*(zeta) potential.

The distribution of ions in the Debye layer is governed by the Poisson–Boltzmann equation^17^:

6 $${c}_{i}\left(0\right)={c}_{oi}exp\left(\frac{{-z}_{i}e\Psi }{kT}\right)$$

where *c*_*i*_(0) is the concentration of ion *i* at the Stern layer, *c*_*oi*_ is the concentration of the ion in the bulk, *z* is the valency, and *e* is the charge on an electron. Counter-ions (those of opposing charge to that of the cell surface) are increased by this expression, whereas co-ions are reduced. The potential arising from the surface potential is given by:

7 $$\psi (r) = \psi e^{ - (\kappa r)} ,$$

where *r* is the distance from the surface and $1/\kappa$ is the thickness of the Debye layer given by

8 $$1/\kappa =\sqrt{\left(\frac{\varepsilon RT}{2cz{F}^{2}}\right)}$$

where *c* is the electrolyte concentration (mol m^−3^). Since the combination of hydrodynamic effects and electrostatic attraction of the closest ions at the surface cause the formation of a stagnant layer around the cell, the value of ψ is difficult to measure. Instead, its effect is expressed through the ζ-potential, defined as the electrical potential at the perimeter of the stagnant layer (known as the slip plane, or hydrodynamic plane of shear). In theory, *ζ* is a product of cellular surface chemistry (principally sialic acid residues). The surface potential at the actual membrane/solution interface will differ from the ζ-potential; however, it is easier to consider the potential at the boundary between the Stern and Debye layers, *ψ*_*st*_, as a fixed point of reference, rather than the actual cell membrane surface potential. The ζ-potential of red blood cells has been studied over many years; Eylar et al.^24^ used it to determine the contribution of sialic acid to surface charge, and Jan and Chien^25^ showed the role of the ζ-potential in cell–cell interactions. Indeed, the ζ-potential is a prime component in the “Erythrocyte Sedimentation Rate” (ESR) assay used clinically to determine the presence of biomarkers of inflammation that later determine erythrocyte-erythrocyte interaction^26,27^.

Understanding the ion concentration at the cell membrane surface is important, since both the GHK equation (Eq. (1)) and the Clausius–Mossotti model (Eq. (2)) assume that the contents of the cell from the inner leaflet of the membrane and across the cytoplasm are electrically homogeneous. Further, use of the Clausius–Mossotti model for cellular analysis typically assumes that cytoplasmic conductivity, *σ*_*cyto*,_ is electrically independent of the cell environment. However, it is known that cells resuspended in a low-conductivity medium rapidly equilibrate with the surrounding medium^28^ and that resuspension of red blood cells causes rapid membrane potential changes^29^, effects rarely accounted for in the model. Furthermore, whilst the Clausius–Mossotti model assumes that cellular compartments are homogeneous, the theory from which the model derives is one of *interfacial* polarisation—that is, it is the electrical properties at the interface between adjacent compartments that are important. The models of membrane conductance (including the surface conductance component) arise because in complex aqueous environments, the ionic behaviour at the interface is substantially different from the bulk.

It is also important to consider that inside the cell, the charges on the *inner* surface of the membrane will cause a second double-layer; this will increase the net ion concentration of the inner surface, and hence raise the calculated value of *σ*_*cyto*_. Previous work^30,31^ showed the importance of considering the inner membrane surface charge. This was developed further by Ingolf Bernhardt and colleagues in their studies of red blood cells^32–34^. They even went as far as altering the GHK equation to account for ion concentrations due to surface effects on both inner and outer membrane surfaces^32^. However, as with the fixed-charge theory, these concepts were largely ignored in favour of a “conventional” GHK model whereby the presence of surface charges were presumed too small to be of significance outside of studies in solutions of low ionic strength.

## Methods

The models in this paper were derived using the data set presented in a prior study^12^ on RBC electrical properties. In summary, data were produced by simultaneous measurement of the ζ-potential via a Malvern Zetasizer Nano (Malvern UK); membrane capacitance and conductance, as well as cytoplasm conductivity by dielectrophoresis^14,35^ using a Deparator 3DEP (Heathfield UK)^33^; and membrane potential, *V*_m_, via the CCCP method, by measuring medium pH (Hanna Instruments, Leighton Buzzard, UK) after treatment with a proton ionophore, a technique that has been widely used for *V*_m_ measurement in RBCs^36^. RBCs were prepared from blood samples taken by venepuncture from four participants, isolated by density gradient centrifugation and entrained for 48 h^15,16^ prior to measurement. Studies were conducted in accordance with the principles of the Declaration of Helsinki, with a favourable ethical opinion from the Research Ethics Committee at the University of Surrey, as described by Henslee et al.^15^. Participants provided written, informed consent after having received a detailed explanation of the study procedures.

Measurements were carried out in an isotonic medium (145 mM NaCl, 7.5 mM KCl: conductivity; 1.8 S m^−1^) or in 10% and 1% dilutions with an isotonic sucrose/glucose solution (conductivities; 0.18 and 0.018 S m^−1^). Measurements in each solution were under five treatments, making 15 conditions overall. Treatments were: control; DMSO solution (0.13%); neuraminidase (final concentration 15 µg/ml); valinomycin (final concentration 30 nM); and neuraminidase + valimomycin. DMSO was used as a solvent for neuraminidase and valinomycin stocks and the final concentration was the same as added with the neuraminidase and valinomycin additions. Neuraminidase was used to remove surface charge at the outer leaflet of the cell membrane; valinomycin as a K^+^ ionophore to increase K^+^ membrane permeability. The surface potential for each treatment was estimated from measurement of the ζ-potential at different conductivities with a Gouy–Chapman model^23^.

Data were analysed by describing linear relationships between parameter sets (either by direct comparison of parameters, or by comparison of parameters following mathematical operations). Relationships were classified according to the square of the correlation coefficient, *R*^2^.

## Results

### Cytoplasm conductivity

**Figure 2:**
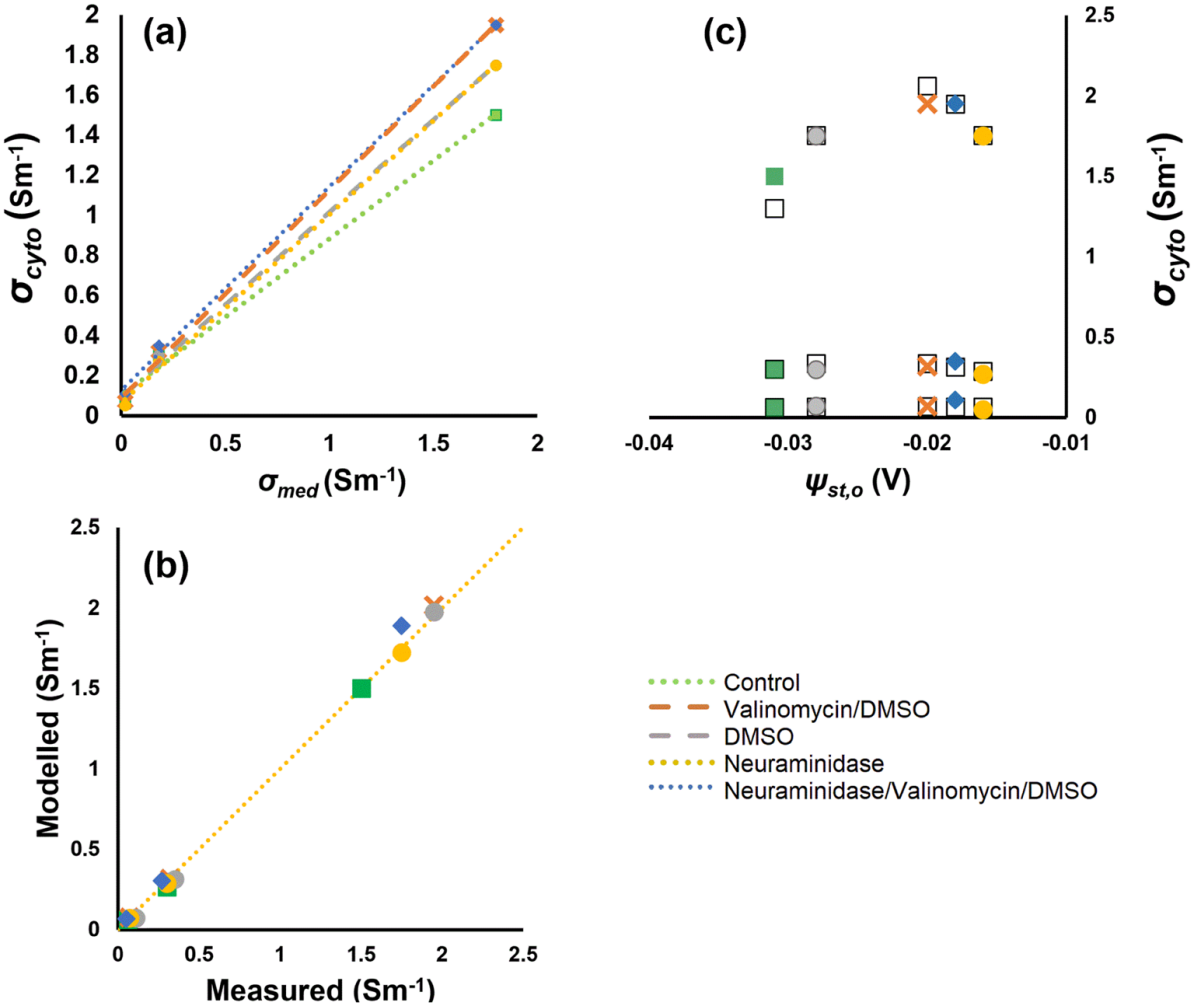
(**a**) A plot of the variation in measured cytoplasm conductivity *σ*_*cyto*_ as a function of medium conductivity (**b**) A comparison of the experimental and predicted values of *σ*_*cyto*_ using the model in Eq. (5). (**c**) Scatter plot of experimental (coloured markers) and predicted (black squares) values of *σ*_*cyto*_ vs. outer membrane surface potential, *ψ*_st_.

A plot of cytoplasm conductivity *σ*_*cyto*_ against medium conductivity *σ*_*med*_ (Fig. 2a) yielded a linear relationship (*R*^2^ > 0.99) irrespective of the five treatment conditions. Best-fit lines were given by:

9 $$\sigma_{cyto} = A \cdot \sigma_{med} + B,$$

with constants* A* and *B* in Table 1*.* The value of *B* (0.096 ± 0.022 S m^−1^) was insensitive to the different conditions, suggesting it may represent the contribution of cytoplasmic components not subject to diffusion or other interactions with the extracellular environment. This could include the conductivity of cytoplasmic components which do not diffuse across the cell membrane, including proteins and ions with low permeability.

**Table 1:** Gradient (*A*), intercept (*B*) and quality of fit (*R*^2^) of linear fits to the variation in *σ*_*cyto*_ with *σ*_*med*_ for the five conditions examined here.

	*A*	*B*	*R*^2^
Control	0.78	0.1	0.995
Val/DMSO	1.03	0.09	0.998
DMSO	1.01	0.13	0.999
Neur	0.92	0.09	0.998
Neur/Val/DMSO	0.94	0.07	0.999

Conversely, the gradient *A* varied with treatment. To interpret the parameter *A*, we first considered the effect of the outer surface charge on an ionic concentration at this face of the cell, *c*_*i*_(0). This deviates from the bulk ion concentration *c*_*b*_ according to the Poisson-Boltzmann equation (Eq. (6)), whereas anionic and cationic concentrations in the bulk are equal. If we consider *Ψ*_*st,o*_ to be the potential at the surface of the outer membrane surface Stern layer; and *z, e, k* and *T* have their usual thermodynamic meanings, then if *A* represents the ratio between free anions and cations in the bulk medium and in the double layer, *A* is given by the expression:

10 $$A= \frac{2}{exp\left(\frac{-ze{\Psi }_{st,o}}{kT}\right)+exp\left(\frac{ze{\Psi }_{st,o}}{kT}\right)}$$

This represents the ratio of the conductivity between bulk and cell surface; that is, the ion concentration on the side of the membrane that faces the extracellular medium (the denominator), as compared to that in the bulk medium (the numerator).

A best-fit value for *Ψ*_*st,o*_ of − 31 mV was previously obtained in control solutions^11^; values under the other conditions described here are available in that work. If we assume that there is ion diffusion across the membrane, then alteration of these ion concentrations will determine the extent of transmembrane diffusion; if cytoplasmic ions equilibrate with the medium, then this expression would represent the ionic strengths across which the cytoplasm would equilibrate.

However, the Clausius–Mossotti factor is concerned with *interfacial* polarisation, so that the conductivity at the *inner* interface, where the membrane meets the cytoplasm, must also be considered. The process in Eq. (10) may be repeated, but now the effect of inner membrane surface charge on cytoplasm conductivity is also considered. Thus, anion and cation concentrations, already distorted from the bulk due to the action of the outer surface charge, are further distorted by the inner Stern layer potential. This was achieved by adapting Eq. (10) to consider the action of a further Poisson-Boltzmann redistribution due to a second surface charge *ψ*_*st,i*_. The resultant expression, Eq. (11), had a best fit for *ψ*_*st,i*_ of − 46 mV:

11 $$A=\frac{2}{exp\left(\frac{-ze({2\Psi }_{st,o}-{\Psi }_{st,i})}{kT}\right) + exp\left(\frac{ze({2\Psi }_{st,o}{-\Psi }_{st,i})}{kT}\right)}$$

**Figure 3:**
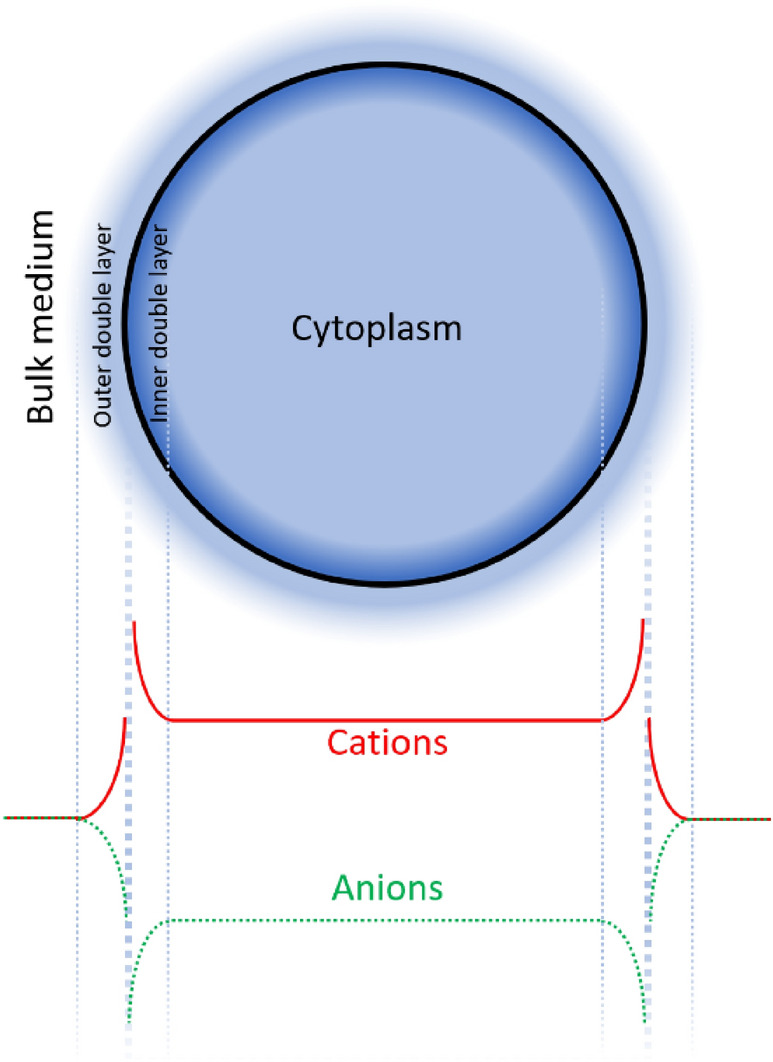
A schematic showing the variation in the concentration of permeable anions and cations across the bulk medium, double-layers and cytoplasm.

A comparison of measured *σ*_*cyto*_ values with those predicted by Eqs. (9) and (11) is shown in Fig. 2b. This formulation gives an average difference between model and measurement of 3.9% when compared to DEP-derived cytoplasm conductivities across all five treatment cases; and at three conductivities of 1.8 S m^−1^, 0.18 S m^−1^ and 0.018 S m^−1^. The effect of the second, inner surface potential can be seen in a plot of the variation of *σ*_*cyto*_ as a function of *ψ*_*st,o*_, as shown in Fig. 2c. If value *A* were based only on the external potential (as per Eq. (7)) we would expect it to form an inverted parabola with a peak at *ψ*_*st,o*_ = 0 mV. However, as a result of the additional term in Eq. (8) we see, in both experiment and model, that the parabola instead peaks at *ψ*_*st,o*_ =  − 23 mV. Collectively, this suggests an anion/cation profile across the membrane similar to that shown schematically in Fig. 3.

This value of *ψ*_*st,i*_ (− 46 mV) suggests that the inner membrane is more polarised than *ψ*_*st,o*_ (− 31 mV^31^). This is consistent with measurement of liposome ζ-potentials^37^ comprising either phosphatidylcholine, PC (which forms the majority of outer membrane leaflet lipids; ζ -potential ≈ − 5 mV) or phosphatidylserine, PS (which forms the majority of inner leaflet lipids; ζ -potential ≈ − 35 mV). An alternative explanation for the difference in polarisation is discussed later.

Of note is that K^+^ is the dominant cytoplasmic cation, whilst Na^+^ is the main extracellular cation. Thus, assuming this relationship is not disrupted in these experiments, the steady-state is formed not by simple cationic diffusion (Cl^−^ permeability in RBCs is high^29^). However, if the distribution of the more permeable cation, K^+^, is due to the establishment of a Donnan state, then charge balance may be maintained even if cations on either side of the membrane are of different species. Collectively, this suggests that when cytoplasm conductivity is estimated by the Clausius–Mossotti factor, what is measured—at least in RBCs—is a combination of outer and inner membrane surface charges, plus a constant, residual value relating to cytoplasmic conductive components. In addition, DEP combined with ζ-potential analysis allows determination of both inner and outer membrane surface charges.

### Membrane potential

The value of *V*_*m*_ is conventionally approximated by the GHK Eq. (2), which has been experimentally tested by measuring it when extracellular permeable ion concentrations are changed, especially in cells with highly polarised membranes such as nerves and muscles^3,38,39^. Here we propose an alternative approach.

**Figure 4:**
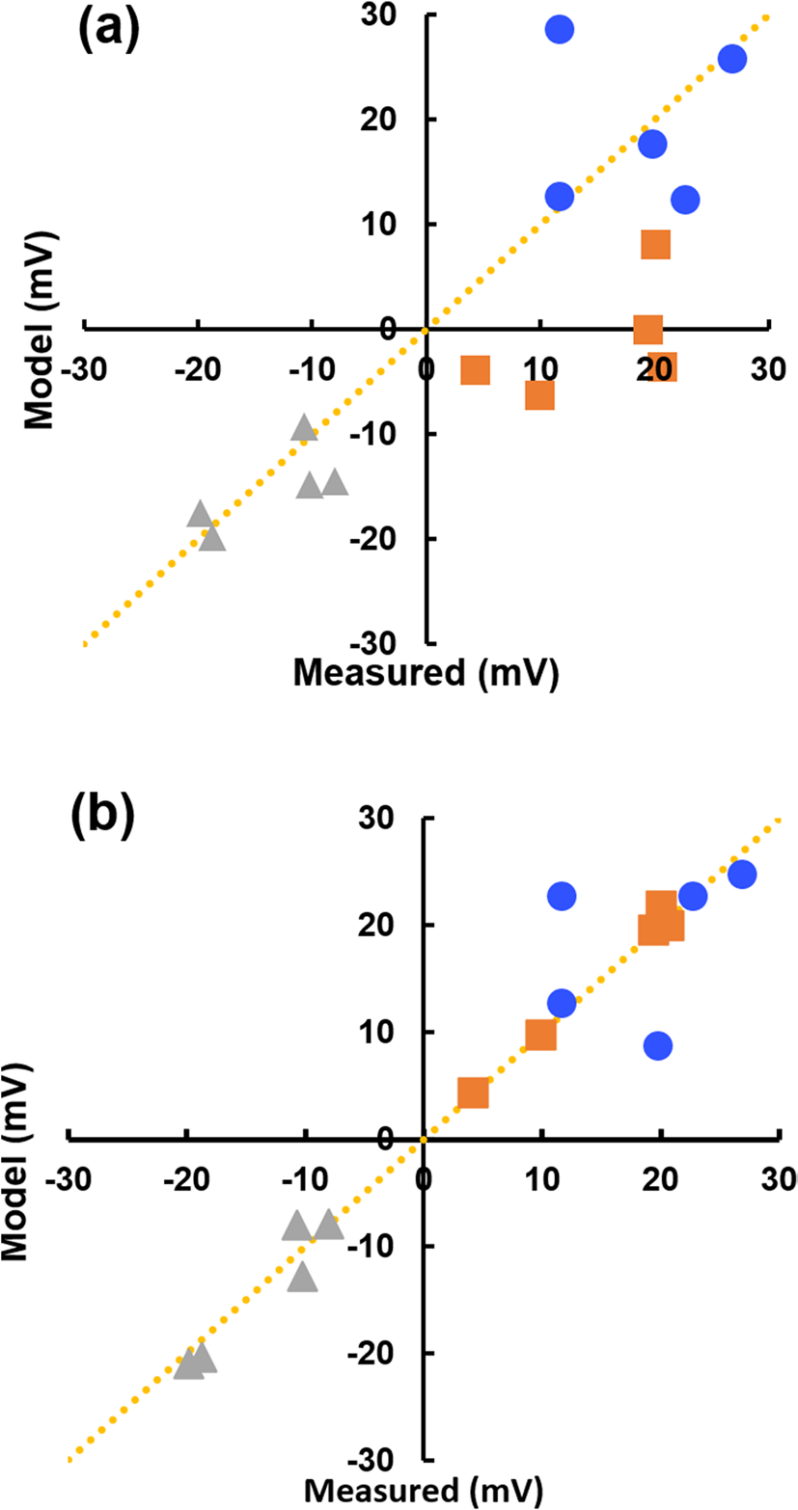
Scatter plots showing the correlation between measured values of membrane potential, and two models of membrane potential: (**a**) Eq. (12); and (**b**) Eq. (13) for measurements derived from normal (triangles; 1.8 S m^−1^) and lower (squares; 0.18 S m^−1^ and circles; 0.018 S m^−1^) conductivities. Dotted line is that of identity.

In the first instance we approximated the potential as calculated by using the equation of Bernstein and Nernst (Eq. 1). To test this, we modelled a logarithmic relationship between *V*_*m*_ and medium conductivity:

12 $${V}_{m}=\frac{RT}{F}{\textit{ln}}\left(\frac{{\sigma }_{cyto} }{{\sigma }_{med}}\right)+{V}_{x},$$

where *V*_x_ is a constant. A comparison of measured and modelled values of *V*_*m*_ using Eq. (12) is shown in Fig. 4a, with reasonable agreement in media of normal (1.8 S m^−1^) and very low (0.018 S m^−1^) conductivity, *σ*_med_—the dotted line is one of identity. However, there was poor agreement with data from experiments in a medium of intermediate conductivity (0.18 S m^−1^). Values of *V*_*x*_ were in the range 21–27.5 mV, except after valinomycin treatments when *V*_*x*_ was 11–12 mV.

An alternative model is one of a significant *linear* relationship between *V*_*m*_ and medium conductivity *σ*_*med*_, which suggests that its origin is something other than by diffusion. One possible origin is the relationship between charge in the cell interior, and the effect of this charge on the transmembrane capacitance. The conventional relationship between voltage *V*, charge *Q* and capacitance *C* (*V* = *Q*/*C*) allows estimation of the effect of anionic and cationic charge imbalances in the inner double layer, compared to the external medium, and the double layer capacitance as derived previously ^12^. This yields the expression:

13 $${V}_{m}= \frac{\left(1/\kappa\right){\sigma }_{med}\Lambda e\left(2-\frac{1}{A}\right)}{2{C}}+{V}_{x}$$

where 1/*κ* is the Debye length; $\Lambda$ the number of ions per m^3^ for a medium conductivity of 1 S m^−1^, and *A* as defined in Eq. (11). *V*_*x*_ was as before. When the results of this equation were compared to the measured values of *V*_m_ (Fig. 4b), a good fit (*R*^2^ = 0.87) was obtained. Only two data points deviated significantly, those for cells in the lowest conductivity medium and treated with neuraminidase. If these are not considered, the fit improved to *R*^2^ = 0.95 (see Discussion). Of the 15 combinations of conductivity and treatments, 40% of *V*_m_ estimates (*n* = 6) were within ± 1 mV of the measured value, 73% (*n* = 11) were within ± 2 mV and all but the two outliers were within ± 3.5 mV.

## Discussion

The *ζ*-potential of a cell fundamentally affects the way in which a cell interacts with extracellular charged entities, from ions to other cells. If the charge at the cell surface is altered, it changes the ratio of anions and cations at the cell surface, changing ion availability for membrane transport; it alters the way in which charged macromolecules, such as antibodies, can approach the surface and bind; and may even alter cell–cell interaction, particularly where cell adhesion is important. Whilst the resting membrane potential has long been studied due to its role in controlling voltage-gated channels, less attention has been focussed on how it may influence how the cell might interact with its environment.

In previous work with RBCs^12^, and subsequently confirmed with platelets^13^, we demonstrated that modulation of *V*_*m*_ alters the ζ-potential. In consequence, developing an understanding of the origin of *V*_*m*_ is essential to understand how this relationship occurs. Furthermore, our previous work with both RBCs and platelets indicated that a linear relationship exists between *V*_*m*_ and *σ*_*cyto*_. Here we have explored how these two parameters vary with medium conductivity *σ*_*med*_ in order to understand the connection between all three parameters, and to apply these insights into understanding the relationship between *V*_*m*_ and *ζ*.

**Figure 5:**
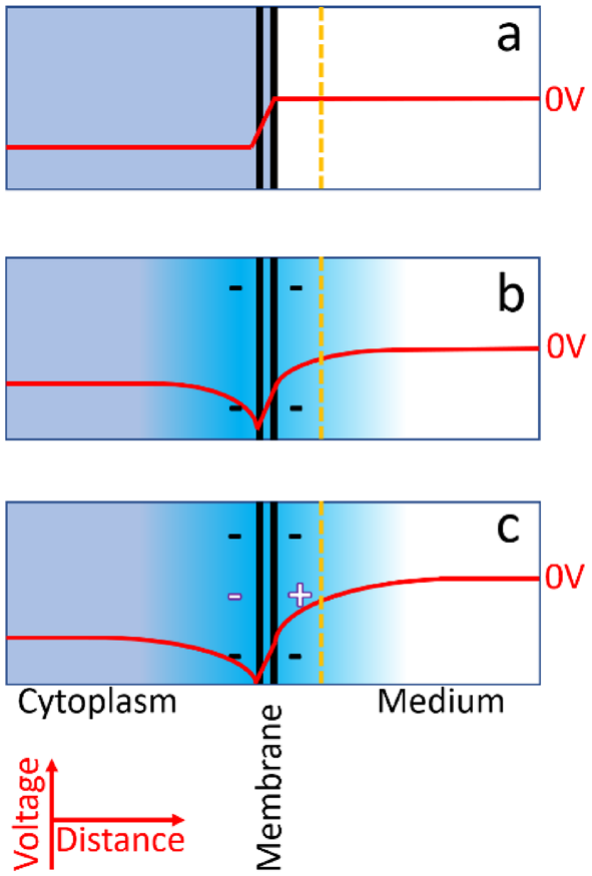
Schematic showing the variation in electrical potential across the membrane (vertical black lines) and shear plane (broken line). (**a**) Potential according to the GHK equation; (**b**) extended to include surface charge effects; (**c**) further extended to include the effects of capacitive charging across the membrane due to the membrane potential.

Both *V*_*m*_ and *σ*_*cyto*_ exhibited a significant dependence on extracellular medium composition (i.e. *σ*_*med*_), consistent with conclusions from DEP measurements that cells, such as RBCs, can leak ions in low-conductivity media^28,32–34^. Our models for both *V*_*m*_ and *σ*_*cyto*_ had a component dependent on the value of *σ*_*med*_ and another that was independent; the *σ*_*med*_-independent component of *V*_m_ was termed *V*_x_. The *σ*_*med*_-dependent component of *V*_m_ showed a linear relationship which suggests that it is not dependent on diffusion, but rather it is Gaussian, caused by a charge imbalance at the inner membrane surface acting across the membrane capacitance to produce a voltage. This would also explain why the membrane potential modulates the RBC *ζ*-potential (Fig. 5). A previous study also found interactions between surface and resting membrane potentials^40^. However, in this case *V*_*m*_ was assumed to be dependent of the surface potential; our model suggests that the inter-relationship is two-way.

This approach to understand the origin of *V*_m_ is not inconsistent with the GHK equation, but suggests a significant second component, at least for red blood cells. In both the logarithmic model of Eq. (12) and the linear model of Eq. (13) the value of the second (*σ*_*med*_-independent) component of *V*_*m*_, termed *V*_*x*_, remained approximately constant; 28–34 mV without valinomycin, and 12–19 mV with valinomycin present. The dependence of *V*_*x*_ on valinomycin (which increases membrane K^+^ permeability) suggests that this component in fact represents the contribution from diffusion, as described by the GHK equation. The constancy of *V*_*x*_ for different values of *σ*_*med*_ suggests that ion permeability remains constant for different values of extracellular ion concentration. Because Eq. (9) shows that *σ*_*cyto*_ (and hence cytoplasmic ion concentration) is proportional to *σ*_*med*_ (and hence medium ion concentration), the membrane potential as calculated by the GHK Eq. (2) would remain constant, as it depends on the *ratio* of interior and exterior ion concentrations. However, it would alter according to the ion permeability of the membrane as seen in the addition of valinomycin. This is consistent with the observed behaviour in *V*_*x*_, though not in the overall variation of *V*_*m*_, suggesting that capacitive and diffusional potentials may each be independent, but significant, components contributing to the overall membrane potential. Only two points did not fit the model; both were treated with neuraminidase and were measured at low conductivity. The datum representing cells treated with valinomycin exhibited a measured *V*_*m*_ greater than that predicted by the model, whilst the reverse was true for that treated with neuraminidase but not valinomycin. Whilst this may be an artefact due to a lack of accuracy of *σ*_*med*_ at low conductivity, the fact that both are treated with neuraminidase (which removes surface charge) suggests that this may alter equilibrium behaviour in low-conductivity solutions (data at higher conductivity remains in line with model predictions). This warrants further study, particularly in light of the different directions of change in the presence or absence of valinomycin.

On a thermodynamic basis, the relationship between *σ*_*med*_ and *σ*_*cyto*_ (Eqs. (9)–(11)) is consistent with the generation of a Donnan steady-state between intracellular and extracellular spaces. Thus, the membrane functions as one that is semipermeable to K^+^ and Cl^−^, but much less so to Na^+^, in the additional presence of impermeable intracellular anionic moieties. An energetic input, the Na-pump, removes inevitable leaks of the relatively impermeant cation into the cell and through this reduction of entropy thus maintains constant cell volume. Moreover, these ion movements potentially only occur within a few nanometres of the membrane, elevating the significance of surface phenomena. It is also notable that the value of *A* (Eq. (9)) is variable and takes values greater than unity in some cases, where the internal surface potential is more than twice that of the external value, making the cytoplasm more conductive than the medium in which the cell is bathed. There have been extensive studies of the capacitive behaviour of lipid membranes (e.g.^41,42^), which evaluated the relationship between surface charge and transmembrane capacitance. However, the situation presented here differs in two important ways; firstly, the compositions of the inner and outer leaflets are different from one another; secondly, the inner leaflet encloses a small space where ion transport can make a significant difference to the intracellular bulk ion concentration. The observation that *σ*_*cyto*_ is lower in RBCs than in many excitable cells also raises the possibility that the inner leaflet Stern potential is lower than that of the outer leaflet. It is often assumed that the reduced intracellular conductivity is due to reduced ionic mobility in a generally more viscous medium, but this suggests an alternative mechanism.

These models have demonstrated how cellular electrical properties and medium compositions might interact. They point to a consistent model of cellular electrical behaviour that explains existing observations, sheds new insight into the origin of the membrane potential and points towards a deeper understanding of how cells might electrically manipulate their immediate environment and how in turn they interact with it.

## Data Availability

Data are available on request from Michael Pycraft Hughes, michael.hughes@ku.ac.ae.
